# Moving toward the Development and Effective Implementation of High-Quality Guidelines in Pediatric Surgery: A Review of the Literature

**DOI:** 10.1055/s-0043-1778020

**Published:** 2024-01-19

**Authors:** Willemijn F.E. Irvine, Olivia K.C. Spivack, Erwin Ista

**Affiliations:** 1 Department of Evidence Based Medicine and Methodology, Qualicura Healthcare Support Agency, Breda, the Netherlands; 2 Department of Pediatric Surgery, Erasmus MC Sophia Children's Hospital, Rotterdam, the Netherlands; 3 Department of Neonatal and Pediatric Intensive Care, Division of Pediatric Intensive Care, Erasmus MC Sophia Children's Hospital, Rotterdam, the Netherlands; 4 Department of Internal Medicine, Division of Nursing Science, Erasmus Medical Center, Rotterdam, the Netherlands

**Keywords:** guideline, methodology, quality, implementation, pediatric surgery

## Abstract

Applying evidence-based guidelines can enhance the quality of patient care. While robust guideline development methodology ensures credibility and validity, methodological variations can impact guideline quality. Besides methodological rigor, effective implementation is crucial for achieving improved health outcomes. This review provides an overview of recent literature pertaining to the development and implementation of guidelines in pediatric surgery. Literature was reviewed to provide an overview of sound guideline development methodologies and approaches to promote effective guideline implementation. Challenges specific to pediatric surgery were highlighted. A search was performed to identify published guidelines relevant to pediatric surgery from 2018 to June 2023, and their quality was collectively appraised using the AGREE II instrument. High-quality guideline development can be promoted by using methodologically sound tools such as the Guidelines 2.0 checklist, the GRADE system, and the AGREE II instrument. While implementation can be promoted during guideline development and post-publication, its effectiveness may be influenced by various factors. Challenges pertinent to pediatric surgery, such as limited evidence and difficulties with outcome selection and heterogeneity, may impact guideline quality and effective implementation. Fifteen guidelines were identified and collectively appraised as suboptimal, with a mean overall AGREE II score of 58%, with applicability being the lowest scoring domain. There are identified challenges and barriers to the development and effective implementation of high-quality guidelines in pediatric surgery. It is valuable to prioritize the identification of adapted, innovative methodological strategies and the use of implementation science to understand and achieve effective guideline implementation.

## Introduction

The movement toward evidence-based medicine began in the 1990s with the goal of establishing a strong empirical basis for the practice of medicine. 1 As part of this movement, there has been an increased emphasis on systematic reviews, meta-analyses, critical appraisal of evidence, and guideline development. 1 Guidelines are advisory documents informed by a systematic review and critical appraisal of the best available evidence, rigorous assessment of the benefits and harms, and additional considerations based on the opinions and experiences of experts and patients. 2 3 These advisory documents are intended to optimize patient care and aim to facilitate more consistent, effective, and efficient medical practice, ultimately leading to improved health outcomes. 4

While sound guideline methodology provides structure to the development process and ensures validity and scientific credibility, 5 there are many challenges to the creation of evidence-based, methodologically sound guidelines, particularly in the field of pediatric surgery. One key challenge is the scarcity of evidence. Due to the rarity of many pediatric surgical conditions, patient numbers are often low, and enrolling patients in trials can pose ethical challenges. Although randomized controlled trials are considered the gold standard in healthcare intervention research, they constitute as little as 0.3% of all available research in the field of pediatric surgery. 6 7 Furthermore, the methodological strategies for guideline development rely on the availability of high-quality evidence. As evidence is often lacking, the methodological strategies employed during guideline development vary widely, in some cases influencing their quality. 8 This situation warrants an evaluation of the current status and quality of guidelines developed in pediatric surgery. Moreover, even in cases where sound guideline methodology is applied, and high-quality evidence is available, achieving reduced practice variation and improved patient outcomes in pediatric surgery remains a challenge without effective guideline implementation. 9

The purpose of this review is to appraise the quality of recent guidelines in the field of pediatric surgery and provide an overview of sound guideline development methodologies and approaches to facilitate effective guideline implementation. Additionally, it seeks to provide recommendations to the pediatric surgical community on how to effectively address challenges to ensure the development and implementation of high-quality care recommendations for the benefit of patients.

## Methodology for the Development and Critical Appraisal of Guidelines

Several types of documents with varying aims and development methods exist to support clinicians in their decision-making process and reduce undesired practice variation. Guidelines are considered the gold standard in this regard. For the purpose of this review, we define guidelines as advisory documents providing recommendations on disease-specific issues that were developed based on a systematic review and critical appraisal of the best available evidence, rigorous assessment of the benefits and harms, and added considerations based on the opinions and experiences of experts and patients. 2 3 However, the term “guideline” is often misused, referring to outputs that lack such specific clinical questions or critical appraisal. The development of guideline recommendations is led by a critical evaluation of the evidence. The generation of specific questions framed by four elements (patient/population, intervention, comparison, and outcomes [PICO]) is a key component to support the systematic review and critical appraisal of the best available evidence. 10 If there is a lack of evidence, clinical consensus statements can be utilized to drive improvements in quality of care. 11 Clinical consensus statements reflect opinions, formulated by subject matter experts, for which consensus is sought using explicit methodology to identify areas of agreement and disagreement. 12

The shift toward evidence-based medicine has stimulated the development of methodology to support the creation of trustworthy guidelines. 1 In 2014, Schünemann et al 13 systematically reviewed published guideline development manuals and guideline methodology reports from governments, ministries, and professional medical societies worldwide (albeit none in the field of pediatric surgery or rare diseases). Their goal was to create an overarching checklist for guideline development: the Guidelines 2.0. This checklist has gained international recognition as the gold standard among methodologists, encompassing 146 items divided into 18 topics. These topics cover all phases of guideline development, including planning and organization, stakeholder involvement, generation of clinical questions, assessment of evidence quality, consideration of outcome significance, wording and formatting of recommendations, and guideline updates. A web link to the complete checklist can be found in the Supplementary Material (available in the online version only).

Evaluating the quality of the evidence base is crucial in modern guideline development, as the outcome of this evaluation aids in assessing the reliability of the evidence and the level of confidence that can be attached to the effect estimates. The methods used to judge the quality of evidence can be dichotomized into judgment per study and judgment of evidence quality for each outcome separately and across studies. The Oxford Center for Evidence-Based Medicine (OCEBM) was the first to develop a model to assess the quality of individual studies, linking the evidence quality to a grade of recommendation (see Table 1 ). 14 The model links judgment of the evidence base linked to study type and study question, typically judged per publication, after which a subjective judgment can be made on how to rate the body of evidence.

**Table 1:** OCEBM levels of evidence and grades of recommendation a

OCEBM levels of evidence b	1	2	3	4	5	
	Systematic review of randomized trials or *n* = 1 trial	Randomized trial or observational study with dramatic effect	Nonrandomized controlled cohort/follow-up	Case series, case–control, or historically controlled studies	Mechanism-based reasoning	
**Grades of recommendation**						
A	Based on consistent level 1 studies					
B	Based on consistent level 2 or 3 studies or extrapolations from level 1 studies					
C	Based on level 4 studies or extrapolations from level 2 or 3 studies					
D	Based on expert opinion or inconclusive/inconsistent studies of any level					

a Adapted from the OCEBM levels of evidence 2011 for treatment questions; different models are available for diagnostic, screening, prevalence, and prognostic questions. 14

b Level may be graded down on the basis of study quality, imprecision, indirectness (study PICO does not match questions PICO), because of inconsistency between studies, or because the absolute effect size is very small; level may be graded up if there is a large or very large effect size.

**Fig. 1:**
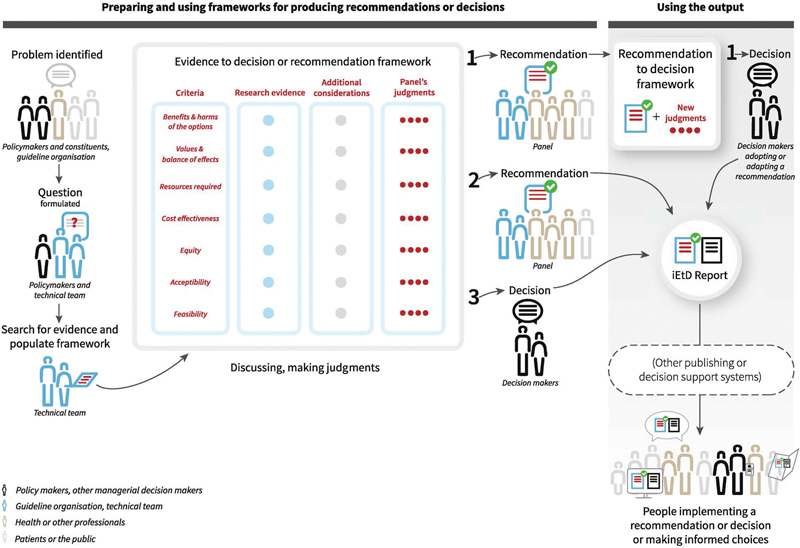
GRADE-EtD frameworks workflow. 16 Referenced article is distributed under the terms of the Creative Commons Attribution 4.0 International License ( http://creativecommons.org/licenses/by/4.0/ ). No changes were made to the figure.

In 2006, the *British Medical Journal* was the first to formally adopt the Grading of Recommendations Assessment, Development and Evaluation (GRADE) system 15 for grading evidence quality by including the use of GRADE in their author instructions for clinical guideline articles. The GRADE system classifies the evidence quality as “high,” “moderate,” “low,” or “very low” and the recommendations as “strong” or “conditional” (see Table 2 ). With the introduction of GRADE, assessing the quality of evidence has become more rigorous and objective. Unlike the OCEBM model, GRADE facilitates the grading of an outcome across studies with explicit criteria for downgrading and upgrading. It also offers a transparent process of moving from evidence to recommendations and a pragmatic interpretation of strong versus conditional recommendations. 15 The GRADE system offers a change in the way evidence quality is assessed, by acknowledging that high-quality evidence does not always lead to strong recommendations and lower quality evidence can, by using a transparent process of moving from evidence to recommendations, support a strong recommendation. In a later phase, the evidence to decision (EtD) framework (see Fig. 1 ) was incorporated into the GRADE system to further provide structure and transparency in the evidence-based decision-making process. 16

**Table 2:** GRADE levels of evidence and strengths of recommendations 15

GRADE	Definition			
High	We are very confident that the true effect lies close to that of the estimate of the effect			
Moderate	We are moderately confident in the effect estimate: the true effect is likely to be close to the estimate of the effect, but there is a possibility that it is substantially different			
Low	Our confidence in the effect estimate is limited: the true effect may be substantially different from the estimate of the effect			
Very low	We have very little confidence in the effect estimate: the true effect is likely to be substantially different from the estimate of effect			
** Types of recommendations a**				
Strong recommendation for the intervention	Conditional recommendation for the intervention	Conditional recommendation for either the intervention or the comparison	Conditional recommendation against the intervention	Strong recommendation against the intervention

a A low probative value of conclusions in the systematic literature analysis does not exclude a strong recommendation in advance, and weak recommendations are also possible with a high probative value. The strength of the recommendation is always determined by weighing all relevant arguments.

While the OCEBM model and the GRADE system are both intended for assessing the quality of evidence, it is important to recognize that they are distinct methodologies. Regrettably, some authors have referred to using the GRADE system when, in fact, they were employing the “grades of recommendations” component of the OCEBM model. Although this confusion may arise from common language, it is undesirable to interchange these terms.

Similar to research articles, guidelines themselves can be appraised to assess their quality. The Appraisal of Guidelines for Research and Evaluation (AGREE) Instrument comprises a set of criteria designed for evaluating the guideline development process and the quality of reporting. 4 In 2010, the AGREE collaboration published an updated version of this instrument: the AGREE II. 17 The AGREE II supports evaluation of a guideline's quality across six distinct domains: scope and purpose, stakeholder involvement, rigor of development, clarity of presentation, applicability, and editorial independence. This evaluation results in an overall quality score, with 1 representing the lowest possible quality, and 7 representing the highest possible quality (see Table 3 ).

**Table 3:** AGREE II for appraisal of guideline quality 17

	Domain	Scoring
1	Scope and purpose	Three items on a 7-point Likert scale
2	Stakeholder involvement	Three items on a 7-point Likert scale
3	Methodological rigor	Eight items on a 7-point Likert scale
4	Clarity of presentation	Three items on a 7-point Likert scale
5	Applicability	Four items on a 7-point Likert scale
6	Editorial independence	Two items on a 7-point Likert scale
Overall	What is your overall impression of the guideline and would you advise to use it?	Score from 1 to 7 with 1 being the worst quality and 7 the best quality

In 2014, Shawyer et al 18 employed the AGREE II instrument in a review on the quality of guidelines published in pediatric surgery journals. Their search yielded 10 eligible documents whose topics ranged from more common issues such as the surgical treatment of gastrointestinal reflux to very rare diseases such as cloacal malformations. The AGREE II quality appraisal indicated a low overall quality of these recommendation documents, as evidenced by a mean total AGREE II score of 18% (SD 5.7%). 18 These documents had the lowest scores, on average, in the domains “rigor of development,” “stakeholder involvement,” and “editorial independence.” This suboptimal methodological quality may be attributed to specific challenges encountered by clinicians in developing pediatric surgical guidelines.

## Challenges for Guideline Development in Pediatric Surgery

The scarcity of evidence for many pediatric surgical conditions poses a key challenge in the development of high-quality guidelines. Where the evidence is scarce, the systematic review, which is an integral part of guideline development, often only yields conclusions based on low to very low-quality evidence. Guideline developers and clinicians involved in the development of guidelines often find it highly challenging to adhere to methodological frameworks such as the GRADE system's evidence-to-decision framework, especially when there is uncertainty regarding the estimated effects on outcomes. In cases where evidence is particularly scarce, discussions may tend toward informal expert consensus. In such instances, developing a clinical consensus statement using an explicit methodology, such as the Delphi method, can serve as a viable alternative, providing increased objectivity. 19

A key feature of the GRADE system is the evaluation of evidence per outcome across studies. Outcomes should be selected a priori and rated for their relative importance to patients. The selection of outcomes for guidelines in pediatric surgery should be based on sound knowledge of the disease and disease trajectory, in addition to children's growth and development. 20 When selecting outcomes to include in a guideline, it is important to acknowledge that treatment effects for pediatric surgical patients may often manifest over extended periods. Selecting appropriate outcomes for congenital malformations can be particularly challenging, as the integrative measurement of treatment effects often necessitates a long–term perspective. Short-term outcomes indicative of longer-term issues may serve as proxies but should be selected carefully. 21 Alternatively, for certain topics included in a guideline, evidence-based judgment can be substituted with decision making through formal clinical consensus.

Another challenge is the high heterogeneity of reported outcomes in available research for many pediatric surgical conditions, such as esophageal atresia, 22 gastroschisis, and Hirschsprung's disease. 23 24 In addition to substantial outcome heterogeneity, a systematic review on investigated outcomes for gastroschisis and Hirschsprung's disease revealed that none of the included studies met all the criteria for transparent outcome reporting. 24 The heterogeneity of outcome reporting adds complexity to the evaluation of evidence across studies, as this impedes overarching comparisons. Rigorous development of disease-specific core outcome sets can greatly assist in overcoming these challenges.

It is noteworthy that the development of high-quality guidelines remains a complex endeavor, even in situations where the aforementioned challenges do not apply. A systematic evaluation of the quality of Japanese guidelines revealed a positive association between higher AGREE II scores and the involvement of a professional methodologist in the guideline development process. 25 In light of this finding, the inclusion or consultation of a guideline methodologist before and/or throughout the guideline development process may lead to a substantial enhancement in quality.

## Available Guidelines and Their Quality

**Fig. 2:**
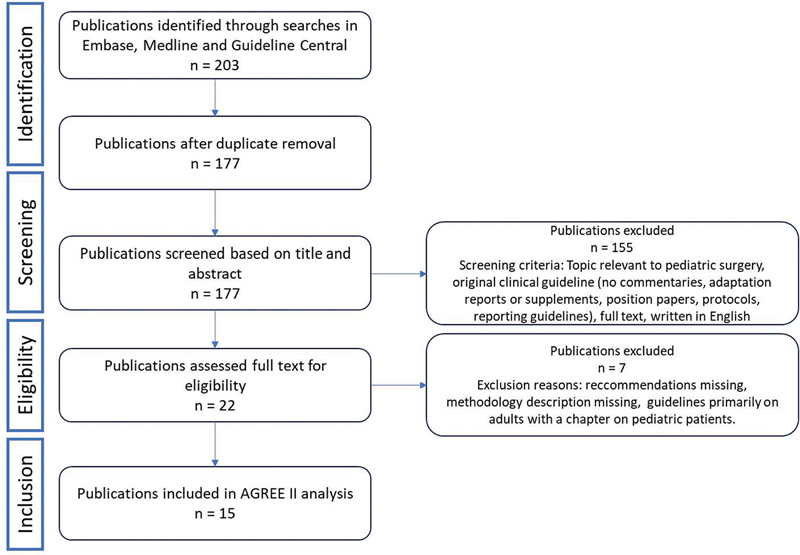
Selection process.

To assess the current status of available guidelines within pediatric surgery, we searched databases (Medline via PubMed, Embase, and Guideline Central) using the terms “Pediatric surgery” and “Guideline” [in title] combined with specific pediatric surgical journals. The full search strategies are available in the Supplementary Material (available in the online version only). As guidelines are preferably up to date, the publication date was limited to the past 5 years (2018 to June 2023). We screened for publications that were referred to as guidelines and that focus on providing clinical recommendations specific to pediatric surgical conditions. Eligible publications had to be available in full text so that their quality could be appraised. For this analysis, other clinical decision tools such as consensus statements, consensus conference reports, and position papers were excluded. Twenty-two potentially eligible publications were identified after screening (see Fig. 2 ), of which 15 were eligible for inclusion. 26 27 28 29 30 31 32 33 34 35 36 37 38 39 40 These 15 guidelines (see Table 4 ) were appraised with the AGREE II instrument by one of the authors. In case of doubt on one of the items, a second author was consulted to judge this item. As the AGREE II incorporates a degree of subjectivity and the aim of this review was to analyze trends in the methodological quality of the identified guidelines on topics within pediatric surgery, mean scores per domain instead of individual scores were reported.

**Table 4:** Included guidelines

Guideline	Developed by	Year	Reference
Surgical practice guidelines for pediatric surgical oncology	International Society of Pediatric Surgical Oncology	2022	26
Neurosurgery guidelines on preventing and managing shunt infection	Indian Society of Pediatric Neurosurgery	2021	27
Guidelines for the management of neonates and infants with hypoplastic left heart syndrome	The European Association for Cardio-Thoracic Surgery and Association for European Pediatric and Congenital Cardiology	2020	28
Pediatric blunt renal trauma practice management guidelines	Eastern Association for the Surgery of Trauma and the Pediatric Trauma Society	2020	29
Guidelines for monitoring and management of pediatric patients before, during, and after sedation for diagnostic and therapeutic procedures	American Academy of Pediatrics and American Academy of Dentistry	2019	30
Foreign body and caustic ingestions in children: a clinical practice guideline	Italian Society of Pediatric Gastroenterology Hepatology and Nutrition and the Italian Association of Hospital Gastroenterologists and Endoscopists	2020	31
Guidelines for perioperative care in neonatal intestinal surgery: Enhanced Recovery After Surgery (ERAS)	ERAS Society	2020	32
Multidisciplinary guidelines for the management of pediatric tracheostomy emergencies	Pediatric Working Party of the National Tracheostomy Safety Project	2018	33
Guidelines for the management of rectosigmoid Hirschsprung's disease	European Reference Network for Inherited and Rare Congenital Anomalies (ERNICA)	2020	34
Guidelines for the management of postoperative soiling in children with Hirschsprung's disease	American Pediatric Surgical Association Hirschsprung Disease Interest Group	2019	35
Guidelines for the management of blunt liver and spleen injuries (updated)	American Pediatric Surgery Association	2023	36
Management of long gap esophageal atresia: a systematic review and evidence-based guidelines	American Pediatric Surgery Association Outcomes and Evidence Based Practice Committee	2018	37
Management of pediatric ulcerative colitis	European Crohn's and Colitis Organization and European Society of Pediatric Gastroenterology, Hepatology and Nutrition	2018	38
Pediatric metabolic and bariatric surgery guidelines	The American Society for Metabolic and Bariatric Surgery Pediatric Committee	2018	39
Diagnosis and management of congenital diaphragmatic hernia: a clinical practice guideline	The Canadian Congenital Diaphragmatic Hernia Collaborative	2018	40

**Fig. 3:**
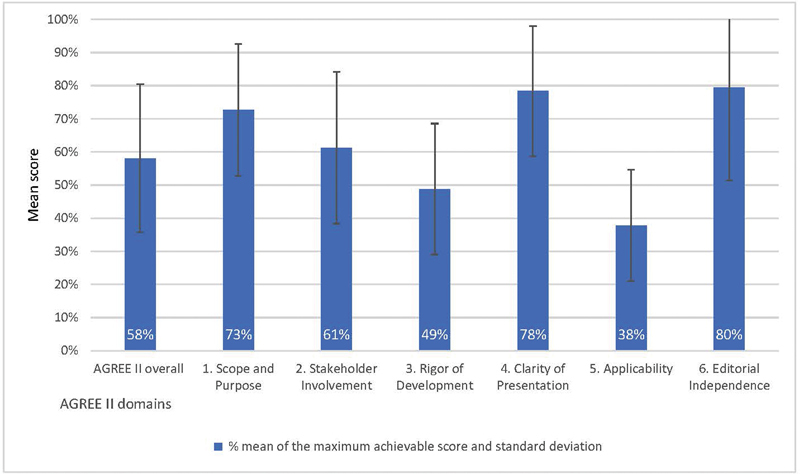
Quality of guidelines in pediatric surgery scored with the AGREE II instrument overall and per domain.

Fig. 3 displays the mean AGREE II scores per domain and provides a collective overall quality score for the guidelines appraised.

The mean overall AGREE II score was 4/7, which represents 58% of the maximum score. Only two individual guidelines scored close to the maximum score in each domain. 29 40 While the appraised guidelines seem to be of higher quality compared with those documents evaluated by Shawyer et al, 18 the overall quality of the sample remains suboptimal. The domain with the lowest average score was applicability (mean score 38%). This suggests that, on average, the evaluated guidelines fell short in accurately describing facilitators and barriers to the guideline's application, potential resource implications, monitoring, and auditing criteria, and how the recommendations could be effectively implemented. The domain “rigor of development” had the second lowest score, with 49%. This was due to many guidelines failing to accurately report strategies for creating recommendations, a lack of clear description of the strengths and weaknesses of the included evidence or no clear link between the recommendations and the supporting evidence. The lowest scoring individual item fell under the scope of the “stakeholder involvement” domain: “The views and preferences of the target population (patients, public, etc.) have been sought.” Only three of the evaluated guidelines reported a strategy for patient or public involvement. 32 34 40 Although there is no standardized approach for patient and public involvement in guideline development, there is increasing recognition of its importance. 41 These findings emphasize the need for rigorous methodology, greater patient involvement, and a focus on applicability in the development of future guidelines in pediatric surgery. Detailed domain scores can be found in Table 5 .

**Table 5:** Mean AGREE II scores for included guidelines 26 27 28 29 30 31 32 33 34 35 36 37 38 39 40

Domain	Overall	Scope and purpose	Stakeholder involvement a	Rigor of development	Clarity of presentation	Applicability b	Editorial independence
Mean domain score	4.07	15.27	12.87	27.33	16.47	10.60	11.13
Max domain score	7.00	21.00	21.00	56.00	21.00	28.00	14.00
% Mean	58	73	61	49	78	38	80

a Lowest scoring individual item: 5. The views and preferences of the target population (patients, public, etc.) have been sought.

b Lowest mean domain score.

## Implementation of Guidelines: The Basic Principles

The availability of a methodologically sound guideline does not in itself result in less practice variation and improved patient outcomes. 42 Where guideline recommendations differ from actual performance, implementation requires a change in healthcare provision. While the challenges of adhering to evidence-based recommendations in clinical practice have long been recognized, there has been a growing emphasis on identifying hindering factors and developing effective implementation strategies to overcome them. 42

The relatively young field of implementation science involves the scientific study of methods that facilitate the implementation of evidence-based practice. 43 This field is guided by theoretical approaches that seek to describe the process of translating evidence into practice (“process models”—often referred to as “process frameworks” in the literature), understand and/or explain implementation outcome-influencing factors (“determinant frameworks,” “classic theories,” and “implementation theories”), and evaluate the successfulness and effectiveness of implementation strategies (“evaluation frameworks”). 44 Drawing on implementation science principles to optimize the uptake of evidence-based practices has been considered of added value to the surgical field. 45 46 The added value to the field of pediatric surgery specifically has been highlighted by Sullivan et al, 9 who also provide an overview of commonly used implementation frameworks and their application purpose.

## Determinants for Guideline Implementation

Implementation found to be successful may not consistently achieve positive impact across settings and contexts; there may be context-specific factors at play that either hinder or facilitate the implementation process. Among an international group of surveyed pediatric surgeons, various barriers to the implementation of new treatment guidelines have been identified, including system, resource, attitudinal, and perceived patient barriers. 47 The most frequently reported barrier in clinical practice was a lack of experience with the newly recommended protocol or procedure. However, pediatric surgeons from underdeveloped countries commonly cited a lack of resources as their primary impediment. Guideline accessibility was reported as the most effective strategy for overcoming identified barriers to implementation, with increased importance for pediatric surgeons from underdeveloped countries. 47 Across the European Union, the availability of guidelines may differ per country. 48 In addition, professional characteristics and organizational and sociopolitical environments have been found to serve as either barriers or facilitators to guideline implementation, 48 which is particularly crucial to consider when developing and implementing European or international guidelines.

The effective implementation of guidelines on topics related to rare pediatric surgical conditions may encounter particular challenges. A recent systematic review of barriers and facilitators to the implementation of guidelines on rare (pediatric and adult) diseases has highlighted various barriers, particularly in the domain of individual health professional factors. These factors include limited awareness of or familiarity with the recommendations and a lack of expertise in the subject area. 49 Healthcare professionals' awareness of or familiarity with a recommendation, as well as their agreement with the recommendation, were identified as key facilitators. 49

## Implementation Strategies

Defined as methods or techniques used to enhance the adoption, implementation, and sustainability of a clinical program or practice, implementation strategies constitute the “how to” component of change initiatives. 50 Accordingly, strategies have been developed to overcome barriers and to increase the pace and effectiveness of implementation. Taxonomies, or classification systems, have been created to describe and organize these implementation strategies, aiming to foster a common understanding and growth of an evidence base for comparative effectiveness. 51 52 53 54 A recently updated version of the Mazza taxonomy 52 53 is considered the most comprehensive and guideline-specific, 55 grouping 51 implementation strategies into six categories: professional (e.g., distribution of guideline material, presentation at meetings), financial (e.g., incentives, grants/allowance, penalties), organizational (e.g., human resources, consumer involvement), structural (e.g., organizational structure, setting/site), regulatory (e.g., legislation, accreditation), and patient/consumer (e.g., printed material, patient education). A comprehensive list of strategies grouped per category according to the updated Mazza taxonomy is provided as Supplementary Material (available in the online version only).

Mapped against this taxonomy, various implementation strategies and combinations have been found to generate positive impact in adult healthcare. 55 However, no single approach has been identified as the key driver of effective guideline implementation. 55 In contrast to adult healthcare, there is currently no systematic review that specifically explores the impact of strategies on guideline implementation in pediatric surgery. A positive impact can be defined as demonstrated improvements in outcomes associated with guideline utilization or impact on the target groups, including patients, families, and healthcare professionals. These improvements may include patient health outcomes, behaviors (e.g., adherence, prescribing practices), knowledge, attitudes, beliefs, and institutional/health system outcomes such as reduced mortality rates or shorter hospital stays. 55

With the recognition that there is indeed no “magic bullet” that could improve professional practice in healthcare, 56 there is a growing emphasis on using implementation science principles to guide the design and tailoring of implementation strategies according to context in a systematic way. 57 While implementation strategies are increasingly selected and tailored using implementation planning approaches (e.g., pre-identified barriers, use of implementation frameworks, stakeholder engagement), a positive impact may still be achieved when such approaches have not been employed. 55 The association between implementation planning and positive impact will be further explored by Peters et al in a future systematic review. 55 We will contact the research team involved to discuss if pediatric surgical studies can be included in this review.

Conducting a process evaluation is essential for comprehending when, where, why, and how certain implementation strategies bring about changes in a particular context, while others fail. 58 While it does not replace outcome evaluation, 59 process evaluation offers a way to evaluate implementation success by exploring the delivery of the planned implementation strategy, the exposure of the target audience, their experiences and opinions, and any factors that may impact the outcomes. 58 Such evaluation fosters iterative adaptation and optimization of implementation strategies. To enhance understanding of effective implementation of both guidelines and consensus statements, sufficient tracking and reporting of implementation strategies in the scientific literature is required. 57

## The Role of Guideline Quality and Development Methods in the Implementation Process

While guidelines do not implement themselves, those with specific characteristics, often referred to as “intrinsic attributes,” may also facilitate effective implementation in clinical practice. In 2015, based on a comprehensive realist literature review, 60 an iterative consensus process, and the engagement of 248 expert stakeholders from 34 countries, the Guideline Implementability to Decision Excellence Model (GUIDE-M) was published. 61 This evidence-based and internationally accepted model presents three core “tactics” influencing guideline implementability, each with subdomains, attributes, sub-attributes, and elements (see Table 6 ).

**Table 6:** The Guideline Implementability Decision Excellence Model (GUIDE-M) a 61

Tactic	Domain	Subdomain	Attribute/sub-attribute (bulleted)/element (parentheses)
Developers of content	Comprehensive	Clinical experts	Multidisciplinary and multijurisdictional
			Researchers and users	
		Target population	Individual patients	
			Family members	
			Groups representing patients	
		Decision makers	Multidisciplinary and multijurisdictional	
			Researchers and users	
		Methodologists	Practice guideline experts	
			Knowledge synthesis experts	
			Health economics experts	
			Ethicists	
			Implementation experts	
	Knowledgeable and credible		
	Competing interests	Financial		
		Professional and/or academic		
		Advocacy		
Creating content	Evidence synthesis	How: execution of methods to develop evidence base	Systematic and reproducible
			Valid and reliable	
		What: completeness of reporting evidence base	Question	
			Eligibility criteria	
			Literature search strategy	
			Critical appraisal	
			Data extraction	
			Data synthesis	
			Reporting	
		When: currency of evidence base		
	Deliberations and contextualization	Clinical applicability	Clinical relevance	
			Patient relevance	
			Implementation relevance	
		Values	Patient/client: • Acceptability • Preferences	
			Provider: • Acceptability • Preferences • Clinical flexibility • Clinical judgment	
			Guideline developer: • Acceptability • Preferences	
			Population/societal: • Acceptability • Preferences • Diversity • Equity	
			Policy: • Acceptability • Preferences	
		Feasibility	Local applicability: • Local adaption • Application tools and strategies	
			Resources: • Availability of resources • Economic evaluation	
			Novelty: • Compatibility • Knowledge and skills	
Communicating content	Language	Simple	Succinct
			Uncomplicated	
		Clear	Actionable • Specific • Unambiguous	
			Effective writing	
		Persuasive	Framing	
			Relative advantage	
	Format	Version	Tailored	
			Modalities • Electronic (dynamic, static) • Non-electronic	
			Document types	
		Components		
		Presentation	Document layout • Visual elements • Length	
			Structure • Match system to the real world • Grouping/ordering	
			Information visualization • Display (tables, algorithms, pictures, graphical display) • Context (framing, vividness, depth of field, evaluability)	

a Referenced article is distributed under the terms of the Creative Commons Attribution 4.0 International License ( http://creativecommons.org/licenses/by/4.0/ ). Minor formatting changes were made to the table for presentation purposes only.

Existing tools designed to improve the development, reporting, and/or evaluation of guidelines have been found to address various components of the GUIDE-M model. 61 Among these tools are those described earlier in this article, such as the AGREE II instrument, 17 the Guidelines 2.0 checklist, 13 and the GRADE system, 15 along with the GuideLine Implementability Appraisal (GLIA) tool, 62 which is specifically tailored to guideline implementation. Efforts to fill gaps in coverage have also begun through the creation of new tools, such as the Guideline Language and Format Instrument (GLAFI) 62 and the Appraisal of Guidelines REsearch and Evaluation-Recommendations Excellence (AGREE-REX) tool. 63 While items in the AGREE II domain “applicability” do attempt to promote implementability, 4 18 this instrument is designed to develop, report, and/or appraise guidelines in their entirety, and it may not always suffice for generating individual recommendations that are both credible and implementable. 64 65 The AGREE-REX tool has demonstrated its validity, reliability, and usability in evaluating specific guideline recommendations, taking into account their clinical applicability, values, preferences, and implementability in a manner complementary to the AGREE II tool. 66

Even with the use of these tools, guideline implementation in the field of pediatric surgery may still be limited by factors inherent to this field, such as the scarcity of evidence and the challenges of outcome selection and outcome heterogeneity. These challenges not only affect the development of high-quality guideline development but also hinder their effective implementation, by constraining fulfillment of the GUIDE-M's “evidence syntheses” domain. In a survey conducted by Lamoshi et al, 47 a majority of pediatric surgeons agreed that treatment guidelines should be evidence-based and expressed a preference for level 1 evidence (evidence of the highest quality) 67 to support the adoption and implementation of a clinical guideline.

Given the limited (yet evolving) evidence base for pediatric surgical conditions, there is a growing need to prioritize the development of disease-specific core outcome sets and recognize the impact of evidence syntheses on the effectiveness of implementation. In this context, process evaluations and iterative implementation approaches are of particular value. 9 Although most surgeons surveyed by Lamoshi et al 47 expressed a preference for level 1 evidence (evidence of the highest quality) 67 to support the adoption and implementation of a clinical guideline, many surgeons were content when the guideline was accepted by their team. The implementation of clinical consensus statements can contribute to narrowing practice variation and improving the quality of care while a high-quality evidence base is being built.

One way to evaluate the implementation of guidelines and generate additional evidence is by using a prospective quality registry. The European Pediatric Surgical Audit (EPSA) 68 funded by the European Reference Network for Rare Inherited Congenital anomalies (ERNICA) is a clinical audit which aims to improve the quality of patient care for rare pediatric surgical conditions. By inputting patient data, participating hospitals can measure and benchmark their quality of care against that of other participating hospitals. Benchmarking not only has the potential to stimulate local improvement initiatives, reduce practice variation, and advance scientific knowledge but also may serve as a feedback mechanism for the standardized measurement of guideline/consensus statement implementation in the field of rare inherited congenital anomalies.

## Conclusions and Recommendations for the Pediatric Surgery Community

The term “guideline” should be employed exclusively if the advisory document has been genuinely crafted following a systematic review and critical appraisal of the best available evidence for specified clinical questions, rigorous assessment of the benefits and harms, and is informed by the insights and experiences of experts and patients. When developed with methodological rigor and implemented effectively, guidelines have the potential to reduce practice variation and improve patient outcomes. Sound methodological approaches exist to structure the guideline development process, and implementation can be promoted throughout the guideline development process and after publication.

The field of pediatric surgery poses specific challenges to the development and implementation of high-quality guidelines, such as the scarcity of evidence and difficulties with outcome selection and outcome heterogeneity. The identification of adapted and innovative methodological strategies that can be applied to address these challenges may be of benefit for the pediatric surgical community. Furthermore, it is advisable to continue conducting high-quality research in the field of pediatric surgery and develop disease-specific core outcome sets. In cases where evidence is very scarce, the systematic and rigorous development and implementation of clinical consensus statements offers an opportunity to reduce undesirable practice variation while building a high-quality evidence base.

The application of the AGREE II instrument as an appraisal tool has highlighted trends such as a lack of applicability and methodological rigor, which impact the quality of guidelines in pediatric surgery. To enhance the quality of guidelines in the future, it is recommended that the AGREE II instrument is not only used for appraisal purposes but also as a tool to oversee and direct the guideline development process. Additionally, to facilitate effective implementation, it is advisable to consider and incorporate the GUIDE-M model into the guideline development process. Tools can be employed to address various components of the GUIDE-M model. Given that the domain *applicability* has been identified as the lowest scoring AGREE II domain for recent pediatric surgical guidelines, the AGREE-REX tool may offer added value. It is further recommended to engage guideline development experts, implementation experts, and representatives of the target population(s), among others, in the process.

Implementation science principles can also help promote the uptake of new guidelines. To gain an understanding of what contributes to successful implementation, it is important to delve deeper into the underlying factors, engage in process evaluation, pursue iterative adaptation, and track and report implementation strategies accordingly. For rare inherited congenital pediatric surgical conditions, the EPSA-ERNICA registry can serve as a standardized method for monitoring the implementation of guidelines and consensus statements.

With recognized barriers to the international implementation of rare disease guidelines and guidelines in pediatric surgery, the scientific study of implementation holds added value for the pediatric surgical community and the patients whom it serves. To maximize its impact, it is essential that those involved have a thorough understanding of the field of study, apply scientific rigor, and keep updated on the evolving literature. Regarding rare inherited and congenital pediatric surgical conditions, the use of implementation science to understand and achieve effective guideline implementation will be prioritized in the upcoming activities of ERNICA.
