# Supplementary material for: Moving toward the Development and Effective Implementation of High-Quality Guidelines in Pediatric Surgery: A Review of the Literature
Source: Eur J Pediatr Surg. 2024 Jan 19;34(2):115–27. doi: 10.1055/s-0043-1778020 (PMC11357791; doi:10.1055/s-0043-1778020)
Supplement: Supplementary file 1 — Supplementary Material [file 10-1055-s-0043-1778020-s2023106759rev.pdf]

## Supplementary Material 1 Guidelines 2.0—Checklist for comprehensive guideline development

https://cebgrade.mcmaster.ca/guidelinechecklistprintable.pdf

## Supplementary Material 2 Search strategies and inclusion

### Embase 110

(“practice guideline”/OR “clinical decision support system”/mj OR (guideline* OR consensus* OR (decision NEAR/3 support NEAR/3 (system* OR tool*))):ti) AND (pediatrics/mj OR “pediatric surgery”/exp/mj OR “congenital disorder”/exp/mj OR (child* OR infan* OR pediatric* OR pediatric* OR congenital*):ti) AND (surgery/mj OR “abdominal surgery”/mj OR “thorax surgery”/mj OR “heart surgery”/mj OR “lung surgery”/mj OR neurosurgery/mj OR “urologic surgery”/mj OR “gynecologic surgery”/mj OR “cancer surgery”/mj OR “orthopedic surgery”/mj OR traumatology/de OR anesthesiology/mj OR embryology/mj OR morphology/mj OR (((surger* OR surgical*) NEAR/3 (abdomin* OR thora* OR neuro* OR urologic* OR gynecologic* OR cancer* OR tumor* OR tumor* OR oncolog* OR orthopedic* OR orthopaedic* OR trauma* OR heart OR cardiac* OR lung OR pulmonar*)) OR neurosurger* OR anesthesiolog* OR traumatolog* OR anesthaesiolog* OR ((child* OR infan* OR pediatr* OR paediatr*) NEAR/3 patholog*) OR embryolog* OR morpholog*):ti) AND [2018–2025]/py

### Medline 65

((((“eur j pediatr surg”[Journal] OR (“european”[All Fields] AND “journal”[All Fields] AND “of”[All Fields] AND “pediatric”[All Fields] AND “surgery”[All Fields]) OR “european journal of pediatric surgery”[All Fields]) OR “Journal of pediatric surgery”[Journal] OR “Seminars in pediatric surgery”[Journal] OR “Pediatric surgery international”[Journal] OR “Orphanet journal of rare diseases”[Journal]) AND “guideline*”[Title]) AND (2018:2023[pdat])

### Guideline Central 28

Search*: Pediatric surgery, within 5 years*

Search date: July 12, 2023

## Supplementary Material 3 Updated Mazza taxonomy[a,1,2]

| |
|---|
| Professional |
| Identify barriers |
| Distribute guideline material |
| Advertise guideline material |
| Present guideline materials at meetings |
| Educate individuals about guideline intent/benefits |
| Educate groups about guideline intent/benefits |
| Recruit an opinion leader who recommends implementation |
| Achieve consensus that guideline should be implemented |
| Provide reminders to individuals/groups about intent/benefits |
| Provide alerts when practice deviates |
| Provide feedback on compliance |
| Provide feedback about patients (outcome data, self-report) |
| Provide feedback from patients |
| Provide feedback from healthcare professionals |
| Print material (summary, algorithm, referral forms, etc.) |
| Tailor guideline |
| Enable self-audit (training, material) |
| Financial |
| Health professional |
| Incentive (individual financial reward or benefit for compliance) |
| Incentive (group or institutional financial reward or benefit) |
| Grant or allowance to individual (not tied to compliance) |
| Grant or allowance to group/institution (not tied to compliance) |

 © 2024. Thieme. All rights reserved.

(*Continued*)

| |
|---|
| Penalty (individual, for non-compliance) |
| Penalty (group/institution, for non-compliance) |
| Change in reimbursement (add/remove/substitute) |
| Patient |
| Incentive (individual financial reward/benefit for compliance) |
| Grant or allowance (not tied to compliance) |
| Penalty (for non-compliance) |
| Incentive (individual non-financial reward/benefit for compliance) |
| Organizational |
| Health professional |
| Additional human resources (number/type) |
| Reallocated or new role |
| Create an implementation/multidisciplinary team |
| Communication between distant health professionals |
| Improve health professional satisfaction (non-financial) |
| Patient |
| Consumer participation in governance |
| Consumer feedback, suggestions, complaints |
| Structural changes |
| Organizational structure (including reorganization) |
| Setting/site of service delivery |
| Physical structure, facilities or equipment |
| Information/communication technology |
| Quality improvement, performance measurement system |
| Method of service delivery |
| Integration of services |
| Risk management provisions (including insurance coverage) |
| Regulatory |
| Legislation or regulation (which enforces or mandates) |
| Ownership or affiliation |
| Licensing, credentialing, or accreditation |
| Patient/consumer |
| Education (single or group) |
| Counseling |
| Group interaction (via social media) |
| Print material (summary, etc.) |
| Reminder |

[a]Referenced articles are published under the terms of the Creative Commons Attribution Licenses (http://creativecommons.org/licenses/by/2.0 and http://creativecommons.org/licenses/by/4.0) respectively. Formatting changes were made to the updated Mazza taxonomy table included in Gagliardi and Alhabib[2] for presentation purposes only.

## References

1 Mazza D, Bairstow P, Buchan H, et al. Refining a taxonomy for guideline implementation: results of an exercise in abstract classification. Implement Sci 2013;8(01):32

2 Gagliardi AR, Alhabib SMembers of Guidelines International Network Implementation Working Group. Trends in guideline implementation: a scoping systematic review. Implement Sci 2015;10(01):54

 © 2024. Thieme. All rights reserved.
